# Phase Correlation Single Channel Continuous Wave Doppler Radar Recognition of Multiple Sources

**DOI:** 10.3390/s22030970

**Published:** 2022-01-26

**Authors:** Khaldoon Ishmael, Yao Zheng, Olga Borić-Lubecke

**Affiliations:** Department of Electrical and Computer Engineering, University of Hawai’i at Manoa, Honolulu, HI 96822, USA; yaozheng@hawaii.edu (Y.Z.); olgabl@hawaii.edu (O.B.-L.)

**Keywords:** biosensors, microwave Doppler radar, multi-subject detection, occupancy sensors, source separation, vital signal processes

## Abstract

Continuous-wave Doppler radar (CWDR) can be used to remotely detect physiological parameters, such as respiration and heart signals. However, detecting and separating multiple targets remains a challenging task for CWDR. While complex transceiver architectures and advanced signal processing algorithms have been demonstrated as effective for multiple target separations in some scenarios, the separation of equidistant sources within a single antenna beam remains a challenge. This paper presents an alternative phase tuning approach that exploits the diversity among target distances and physiological parameters for multi-target detection. The design utilizes a voltage-controlled analog phase shifter to manipulate the phase correlation of the CWDR and thus create different signal mixtures from the multiple targets, then separates them in the frequency domain by suppressing individual signals sequentially. We implemented the phase correlation system based on a 2.4 GHz single-channel CWDR and evaluated it against multiple mechanical and human targets. The experimental results demonstrated successful separation of nearly equidistant targets within an antenna beam, equivalent to separating physiological signals of two people seated shoulder to shoulder.

## 1. Introduction

Since it was theorized in 1842, the Doppler effect has been used in the context of radar sensing to detect moving objects from stationary backgrounds in a variety of areas, ranging from cosmology to meteorology. Small-size and low-power Doppler radars have also been used in medical and search and rescue applications to provide non-invasive and unobtrusive diagnoses of cardiopulmonary conditions [1,2]. Recent work on physiological Doppler radar further sought to extend continuous radar monitoring beyond controlled settings and into unconstrained environments common to applications such as security, human–machine interface, at-home medical tests, smart buildings and walking aids [3,4,5].

The challenge to enable radar monitoring within unconstrained environments is that there could be many targets within the radar’s scope, and nearby individuals reflected signals superimpose, creating a signal mixture consisting of multiple physiological motions [6,7]. Earlier research pursued to separate the signal mixture using complex transceiver architectures or advanced signal processing algorithms. In [8,9], Borić-Lubecke et al., and Lee et al., demonstrated the feasibility to separate multiple spectral diverse or spatially diverse cardiovascular-related motions with single-antenna and multiple-antenna CWDRs. In [10], Rivera et al., presented a multi-target detection method for heart and respiration rates, which applies clustering and multiple signal classification (MUSIC) algorithms to ultra-wideband (UWB) radar output. In [11,12], Fadel et al., and Cardillo et al., step frequency CW (SFCW) radar and MIMO architectures have also been proposed for multi-target detection and tracking [13,14].

Albeit effective, hardware-intensive, or processing-intensive methods for multi-target separation are often costly. For instance, UWB radars require high-speed analog to digital converters (ADCs), and FMCW radars require calibration to compensate for the non-linearity during frequency sweeping. MUSIC algorithm involves singular value decomposition (SVD) with near cubic complexity. Therefore, these methods are challenging to implement on low-power, computation-limited radar sensors standards for physiological monitoring.

To reduce the implementation cost, this work proposes a simple, single-channel CWDR method for multi-target detection using phase sweeping. Conceptually, the detection accuracy of a single-channel CWDR of a single target varies by the target range, due to issues known as null and optimal points [15]. A null point occurs when the round trip distance is even multiples of λ/8, which minimizes the detection sensitivity. An optimal point occurs when the round trip distance is odd multiples of λ/8, which maximizes the detection sensitivity. If a single target is present in a radar field of view, phase tuning can be used to optimize detection sensitivity [16,17,18]. In a multiple target scenario, phase tuning provides different phase correlation and thus signal mixtures from the multiple targets that can be further separated in frequency and time domains. Assuming the targets are not at the same range, we may change the initial phase offset of the radar signal to manipulate the null and optimal points and “amplify” the physiological signal of targets at optimal points while “suppressing" the targets at the null points. Given that each human has distinguished anatomy and distinguished physiological signals, both in shape and frequency, signals of individuals can be separated and identified using simple signal processing algorithms such as Fast Fourier transform (FFTs).

Our prior work [19,20] demonstrated the phase correlation detection principle for multiple targets using range discrimination by adjusting target position in multiples of λ/8. However, adjusting the target range may not always be possible nor practical. This work provides in-depth analysis and full implementation of phase sweeping CWDR system multi-target detection and extensive evaluation results. The main contributions of this work are highlighted in the following: (1) We presented a theoretical analysis of the proposed single-antenna single-channel phase sweep method for multi-target detection with individuals at similar nominal ranges. (2) We evaluated the performance of the phase sweep method via software simulation, robotic movers, and human subjects. The results demonstrated that our system could monitor nearly equidistant subjects seated shoulder to shoulder without changing range, beam angle, or applying other more complex signal separation techniques.

## 2. Theoretical Analysis

Doppler theory states a target with a time-varying position with no net velocity will reflect the transmitted signal with different phases in proportion to the time-varying position of the target. In respiration detection using CWDR, the occurrence of Doppler shift is caused by the displacement of the chest, and this effect can be observed from the phase change of the modulated received signal. The transmit signal T(t) from the CWDR can be expressed as:

(1) $$T\left(t\right)=A\;cos\left(2\pi ft+\phi \left(t\right)\right)$$

where *A* is the transmitted signal amplitude, *f* is the oscillation frequency, *t* is the elapsed time, and $\phi \left(t\right)$ is the initial phase noise of the transmit signal. The transmitted signal is reflected by *N* multiple subjects, at a nominal distance $d_{0N}$ with time-varying displacement $x_{N}\left(t\right)$. The received signal $R_{N}\left(t\right)$, can be expressed as:

(2) $$R_{N}\left(t\right)=A_{N}\;cos\left[2\pi ft+\frac{4\pi \;d_{0N}}{\lambda }+\frac{4\pi \;x_{N}\left(t\right)}{\lambda }+\Delta \phi_{N}\left(t-\frac{2\;d_{0N}}{c}\right)\right]$$

where $A_{N}$ is the received signal amplitude, λ is the wavelength of the signals and $\Delta \phi_{N}\left(t-\frac{2\;d_{0N}}{c}\right)$ is the total time-delayed version of the signal source phase noise. The total reflected phase shift $\theta_{N}$ includes phase change at the target surface $\theta_{N0}$ and phase delay due to target range $d_{0N}$:

(3) $$\theta_{N}=\frac{4\pi \;d_{N}\left(t\right)}{c}+\theta_{0N}$$

Due to each person’s complexity and unique features of human torso anatomy, the actual target range $d_{0N}$ will be slightly different for different individuals at the same nominal range, which causes differences in the phase shifts among targets. After the received signal is mixed with the local oscillator (LO) signal derived from the same source as the transmit signal, and higher frequency components are filtered, mixer output produces a baseband signal that can be expressed as $B_{N}\left(t\right)$:

(4) $$B_{N}\left(t\right)=A_{N}\;cos\left[\theta_{N}+\frac{4\pi \;x_{N}\left(t\right)}{\lambda }+\Delta \phi_{N}\left(t-\frac{2\;d_{0N}}{c}\right)\right]$$

Changing the total reflected signal phase in Equation (3) is possible, by introducing voltage tunable phase shift $\theta_{ps}$ in the received signal path resulting in:

(5) $$R_{N}\left(t\right)=A_{N}\;cos\left[2\pi ft+\frac{4\pi \;x_{N}\left(t\right)}{\lambda }+\Delta \phi_{N}\left(t-\frac{2\;d_{0N}}{c}\right)+\left(\theta_{N}+\theta_{ps}\right)\right]$$

At the mixer output, baseband signals including this phase shift $\theta_{ps}$ can be expressed as:

(6) $$B_{N}\left(t\right)=A_{N}\;cos\left[\frac{4\pi \;x_{N}\left(t\right)}{\lambda }+\Delta \phi_{N}\left(t-\frac{2\;d_{0N}}{c}\right)+\left(\theta_{N}+\theta_{ps}\right)\right]$$

From Equation (6), we can have two approximations. The first one is an optimum point [17] when the value of $\left(\theta_{N}+\theta_{ps}\right)$, is an odd multiple of π/2 and target oscillation amplitude is small compared to the wavelength $x\left(t\right)\ll \lambda$. Assuming that residual phase noise is small, the baseband signal can be approximated as:

(7) $$B_{N}\left(t\right)=\frac{4\pi \;x_{N}\left(t\right)}{\lambda }$$

Hence, the baseband signal is now linearly proportional to target displacement. The second approximation is the null point when the value of $\left(\theta_{N}+\theta_{ps}\right)$ is an even multiple of π/2, and the baseband signal can be approximated as:

(8) $$B_{N}\left(t\right)={\left[1-\frac{4\pi \;x_{N}\left(t\right)}{\lambda }\right]}^{2}$$

In this case, the output signal is no longer linearly proportional to target displacement resulting in reduced fundamental frequency content. The square term results in signal distortion by doubling the signal frequency. Therefore, significant fundamental suppression and an increase in second harmonic content can identify the null point. The phase shift of π/2 shifts the target from null to the optimum position, and the phase shift of π is sufficient to ensure that each target would sweep through at least one null and one optimum position. By tuning the $\theta_{ps}$, the total phase shift can be adjusted to amplify or suppress $B_{N}\left(t\right)$ signals sequentially from multiple sources and allow sensing multiple subjects within a single antenna beam width. This method can be performed without beam steering or other more complex signal separation techniques. Since human subjects are unlikely to present the exact same refection surface configuration at the same actual distance, this method holds promise for distinguishing closely spaced, nearly equidistant individuals. Simulation and experimental results that confirm the effectiveness of this method will be presented in the following sections.

## 3. Simulation

**Figure 1:**
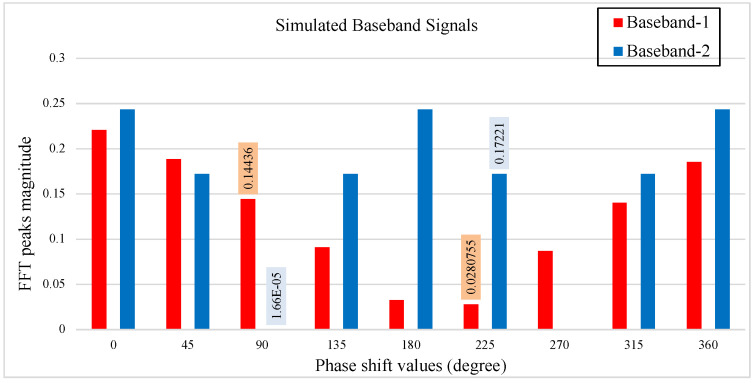
The figure shows two simulated receiving signals, 0.2 Hz baseband-1 and 0.3 Hz baseband-2, with phase sweeping between $\theta_{ps}=0^{\circ }$ to $\theta_{ps}=360^{\circ }$, incremented at $\Delta \theta_{ps}=45^{\circ }$ per step.

We evaluated the performance of the phase sweep method via software simulation to test the feasibility of separating multiple sources. The baseband received signals with phase shift value in the time domain were generated using Matlab. The baseband signal in the radar system is a function of the radar wavelength, fixed displacement of the targets and variable phase shift in Equation (4). In this simulation, two signals with frequencies 0.2 Hz and 0.3 Hz and a small phase offset between them represent respiration signals from two human subjects. The two signals start with a phase shift value $\theta_{ps}=0^{\circ }$, where the phase of the baseband signals spectrum is at arbitrary peaks. The suppression of $B_{N}\left(t\right)$ signals, occurred when the total value $\left(\theta_{N}+\theta_{ps}\right)$ in the baseband signals is an even multiple of π/2. We tuned the phase shift values to suppress each signal to demonstrate the multiple source separation using the phase sweep technique. Figure 1 shows that source 2 is suppressed at $90^{\circ }$, while source 1 is suppressed at $225^{\circ }$.

**Figure 2:**
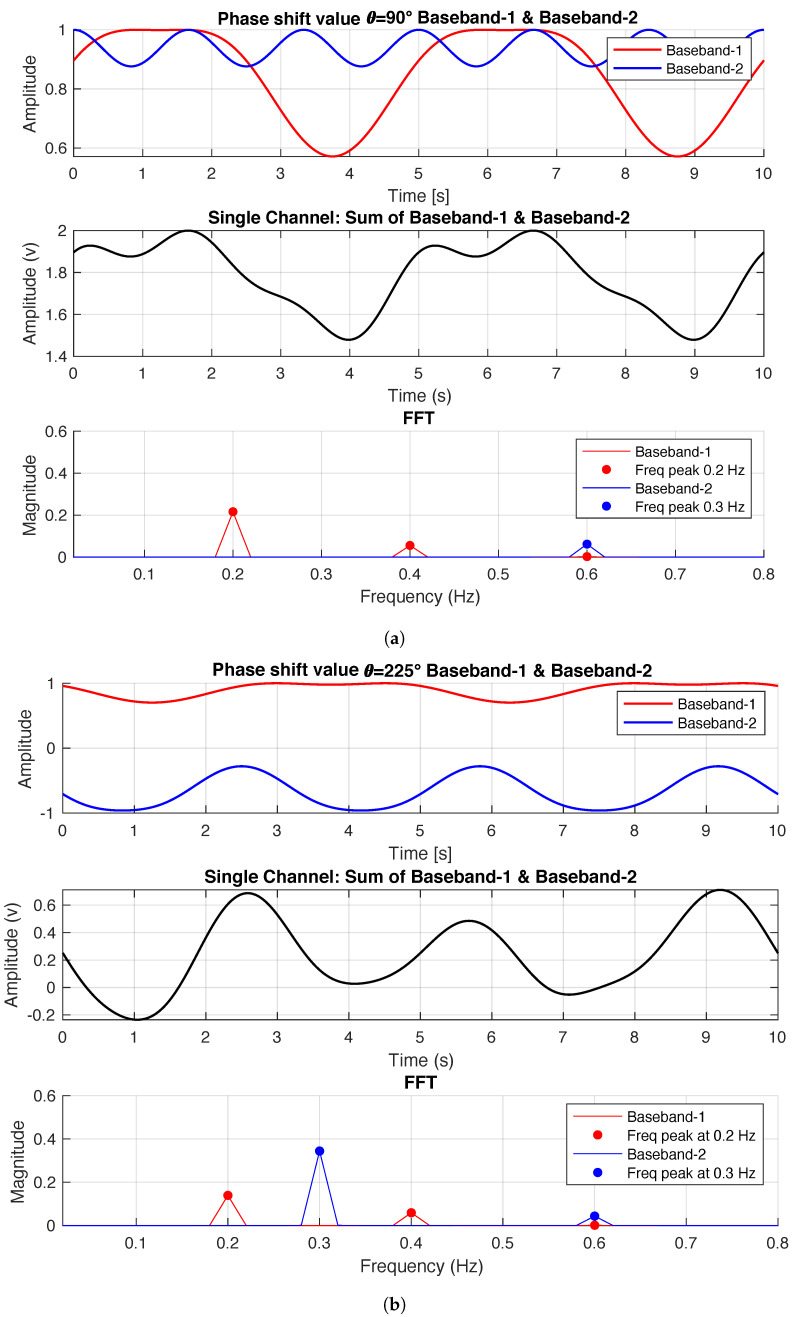
The figure shows two simulated baseband received signals, 0.2 Hz baseband-1 and 0.3 Hz baseband-2. Baseband-1 is at an optimum point, and baseband-2 is at a null point, when $\theta_{ps}=90^{\circ }$ (**a**). Baseband-1 is at a null point, and baseband-2 is at an optimal point, when $\theta_{ps}=225^{\circ }$ (**b**).

Figure 2 shows the simulated baseband signals in the time and frequency domains with phase shift $\theta_{ps}=90^{\circ }$ in Figure 2a, and $\theta_{ps}=225^{\circ }$ in Figure 2b. The oscillation frequencies of each source will be observable as distinct peaks in the frequency spectrum of the composite signal. The phase shift sweep between multiple baseband signals goes from optimum to null point and vice versa approximately every π/2. The simulation results agree with null/optimum theory, where the distance between an optimum and adjacent null point is π/2. Detecting the changes in FFT magnitude and comparing with our statement of which target’s signal has gone through null points due to the difference in the phase shift, we can then uniquely distinguish each signal source in the single-channel single antenna radar system.

## 4. Implementation and Experiments

**Figure 3:**
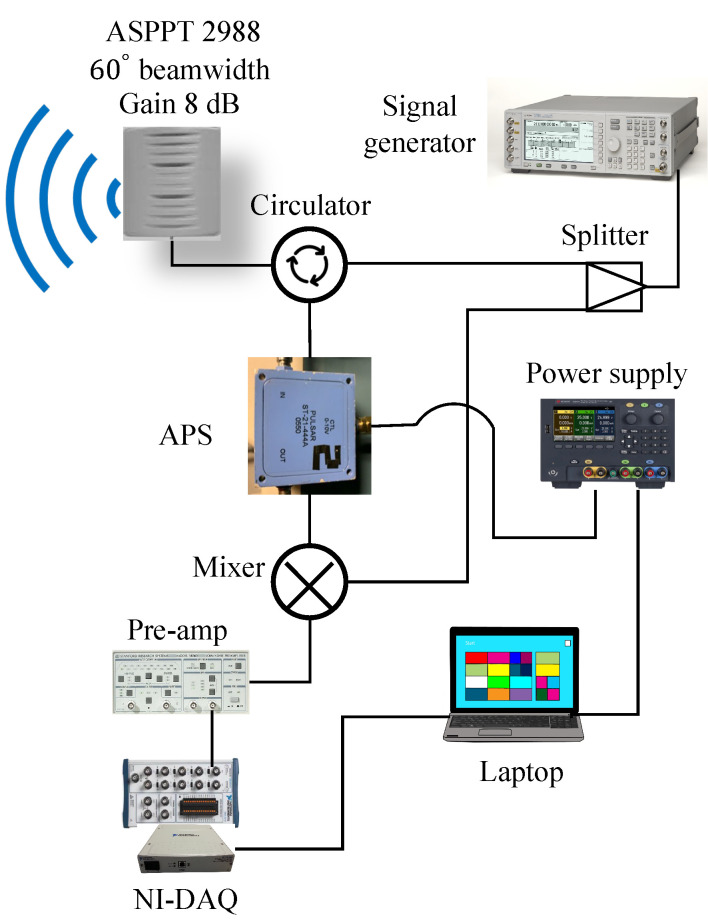
The figure shows a block diagram of phase-sweeping single-channel single-antenna radar system operating at 2.4 GHz. The analog phase shifter (APS) is installed in the radar’s received path and is controlled via a programmable power supply.

A single-channel radar was assembled using ASPPT 2988 from Antenna Specialist^™^ antenna having 8 dBi gain and $60^{\circ }$ E plane beam width, Agilent E4438C signal generator, coaxial components, RF-Lambda RFLC-301-4S circulator, and Mini-Circuits ZFSC 2–2500 splitter and ZFM-4212+ mixer. Using the LabView program, a computer recorded the output signal from the radar system, which was fed to the low-pass filter preamplifier Stanford Research SR560 with a cut-off frequency of 10 Hz and $10^{2}$ gain. The DC offset was compensated to avoid amplifier saturation. The baseband data obtained from the radar system was sampled and recorded using a DAQ NIUSB-6281 at a rate of 100 Hz. Analog voltage tunable phase shifter PULSAR ST-21-444A was inserted in the received signal path preceding the mixer in Figure 3. Phase shifter voltage was controlled with a programmable power supply Keysight E36312A.

**Figure 4:**
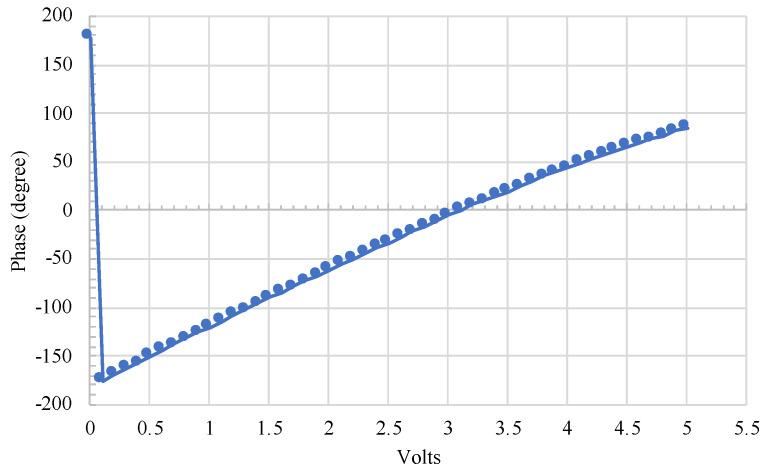
The phase shifter’s output as a linear function of applied voltage.

The analog phase shifter APS has a frequency range of 2 GHz–2.6 GHz, insertion loss of 5 dB max, phase shift $\theta_{ps}=360^{\circ }$ and voltage value ranging 0–10 v. A vector network analyzer was used to characterize the analog phase shifter. Figure 4 shows the measured phase shift for voltage sweep up to 5 v, corresponding to phase shift from $-178^{\circ }$ to $82^{\circ }$. This phase range is more than sufficient to ensure that each target would sweep through at least one null and one optimum position.

### 4.1. Robotic Mover Detection

**Figure 5:**
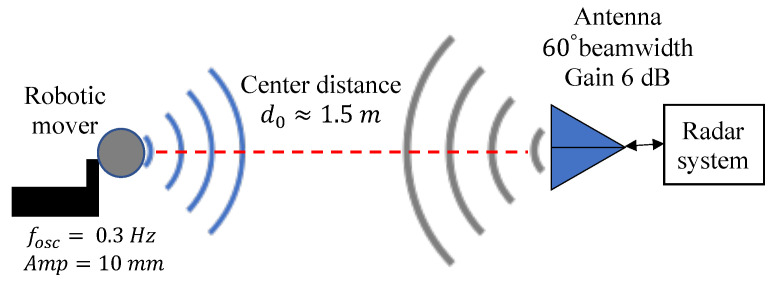
The figure shows the experiment setup with the phase-sweeping radar and one robotic mover. The distance between the radar and the mover is $d_{0}$ = 1.5 m. The mover oscillates at 0.3 Hz frequency and 10 mm amplitude.

We used robotic movers to simulate repetitive respiration motions to obtain reproducible results before testing the system with human subjects. Under normal conditions, the human respiratory rate is between 12 and 18 breaths per minute, corresponding to 0.2–0.3 Hz with maximum chest displacement of 10 mm peak-to-peak [21]. This respiration model was simulated using robotic movers. The reflector was a metallic target attached to a translation stage with one motion axis. Target one is a spherical shape of about 15 cm diameter. Target two is a square plate with dimensions of 20 cm × 15 cm, with each target was mounted on a Griffin Motion LNS-100 Series Linear Stage with a Galil DMC30010 controller. The stage of each target consists of a mount actuated via stepper drive controlled via serial interface, which permits automated movement sequences position resolution within 1 μm, measured as commanded position versus reported position. The single robotic mover was tested initially to demonstrate phase correlation experimentally as illustrated in Figure 5. The spherical robotic mover was placed within the antenna beam, at distance $d_{0}$ = 1.5 m from the center point to the radar antenna. The robotic mover was oscillating at a frequency of 0.3 Hz with a peak-to-peak amplitude of 10 mm.

**Figure 6:**
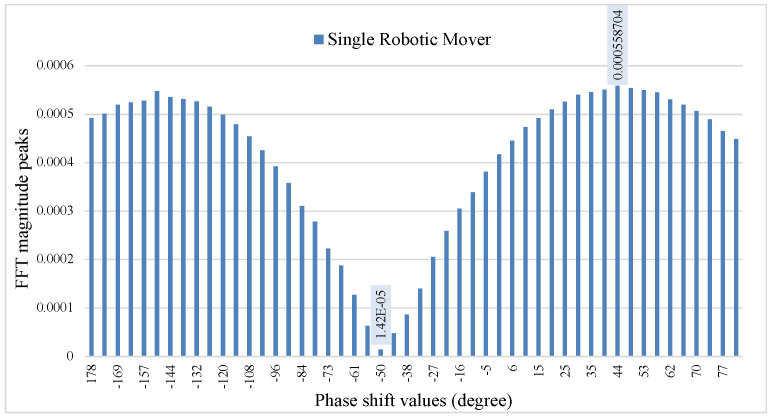
The figure shows the demodulated signal, with phase sweeping between $\theta_{ps}=-178^{\circ }$ to $\theta_{ps}=82^{\circ }$. The robotic mover 0.3 Hz is at a null point when $\theta_{ps}=-50^{\circ }$ and at an optimum point when $\theta_{ps}=44^{\circ }$.

**Figure 7:**
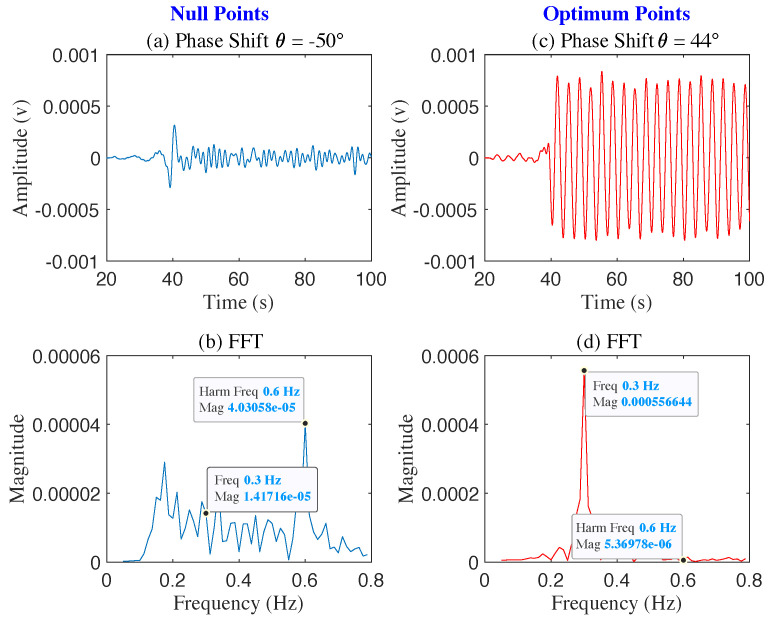
The figure shows two sets of demodulated signal, with $\theta_{ps}=-50^{\circ }$ (**a**,**b**) and $\theta_{ps}=44^{\circ }$ (**c**,**d**). The robotic mover 0.3 Hz is at a null point when $\theta_{ps}=-50^{\circ }$, as the first harmonic 0.6 Hz surpassing the fundamental 0.3 Hz (**b**), and at an optimum point when $\theta_{ps}=44^{\circ }$, as the fundamental surpassing the first harmonic (**d**).

Figure 6 shows the FFT of the output signal for the phase shift sweep from $-178^{\circ }$ to $82^{\circ }$. At the phase shift value $\theta_{ps}=-50^{\circ }$ the robotic mover’s signal was suppressed (null position), whereas phase shift value $\theta_{ps}=44^{\circ }$ shifted the robotic mover’s signal to the optimum position. Figure 7 shows time and frequency domain signals for $\theta_{ps}=-50^{\circ }$ and $\theta_{ps}=44^{\circ }$ At the phase shifter voltage value 2.2 V. corresponding to phase angle $=-50^{\circ }$, the fundamental of the baseband signal is suppressed, and the second harmonic is visible. In contrast, at a phase angle of $44^{\circ }$, the fundamental signal amplitude is maximum, and the second harmonic is absent. The offset phase shift value in the baseband signal goes from optimum to null point and vice versa approximately every π/2, making the baseband signal in the optimum point position at phase angle $44^{\circ }$, for phase shifter voltage of 4 v. The results agree with the simulation and null/optimum theory, where the distance between an optimum and adjacent null point is π/2.

### 4.2. Two Robotic Targets Detection

**Figure 8:**
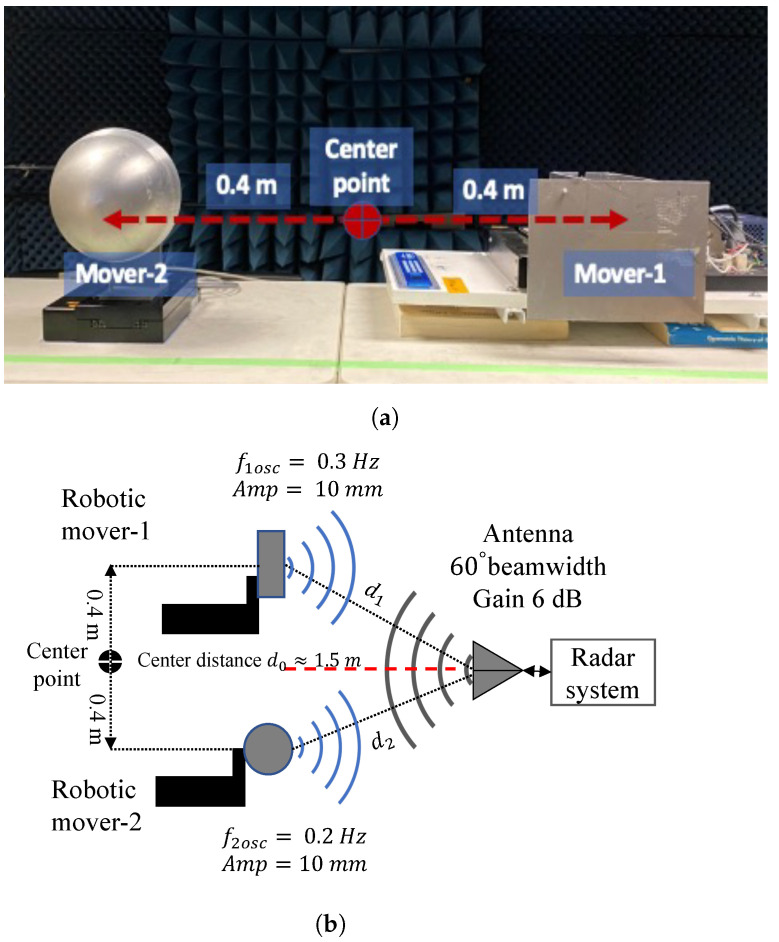
Photograph of the two robotic movers placed facing radar antenna (**a**). The figure shows the experiment setup with the phase-sweeping radar and two robotic movers (**b**). The nominal distance between the radar and the movers is $d_{0}$ = 1.5 m. The distance between the movers is 0.8 m. Mover-1 oscillates at 0.3 Hz frequency and 10 mm amplitude. Mover-2 oscillates at 0.2 Hz frequency and 10 mm amplitude.

**Figure 9:**
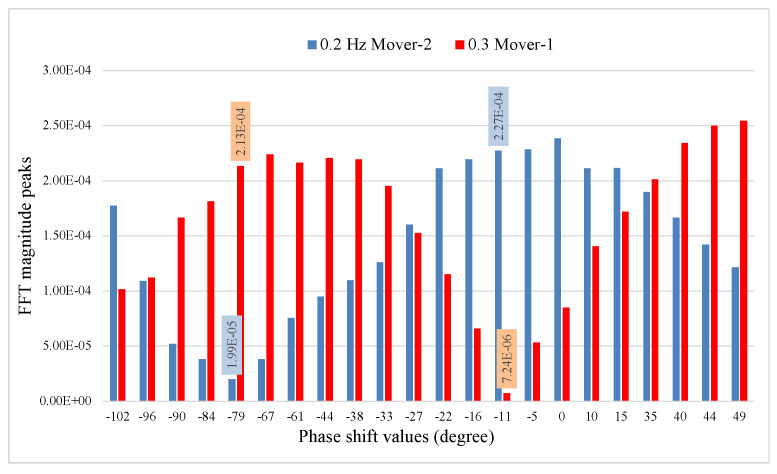
The figure shows the demodulated signals, with phase sweeping between $-102^{\circ }$ to $49^{\circ }$. Mover-1 at 0.3 Hz is at an optimum point, and mover-2 at 0.2 Hz is at a null point when $\theta_{ps}=-79^{\circ }$. Mover-1 is at a null point, and mover-2 is at an optimum point when $\theta_{ps}=-11^{\circ }$.

Two robotic movers were placed facing the radar antenna at a distance $d_{0}=1.5$ m, as illustrated in Figure 8. The center-to-center distance between the robotics movers was 0.8 m, simulating the distance between two people seated shoulder to shoulder. Figure 8a shows the photograph of the two robotic movers placed facing radar antenna at a distance $d_{0}=1.5$ m (a) The experimental layout of the radar system with mover-2 oscillating at 0.2 Hz and mover-1 oscillating at 0.3 Hz is illustrated in Figure 8b. The FFT of the baseband signal in Figure 9 show the output of the phase shift values from $-102^{\circ }$ to $49^{\circ }$. At phase shifter angle $-79^{\circ }$, the baseband signal of 0.2 Hz mover-2 is suppressed to the null point, and 0.3 Hz mover-1 shifted to the optimum point. At the phase shifter angle $-11^{\circ }$, the baseband signal of 0.2 Hz mover-2 is in the optimum point, and 0.3 Hz mover-1 is suppressed to the null point.

**Figure 10:**
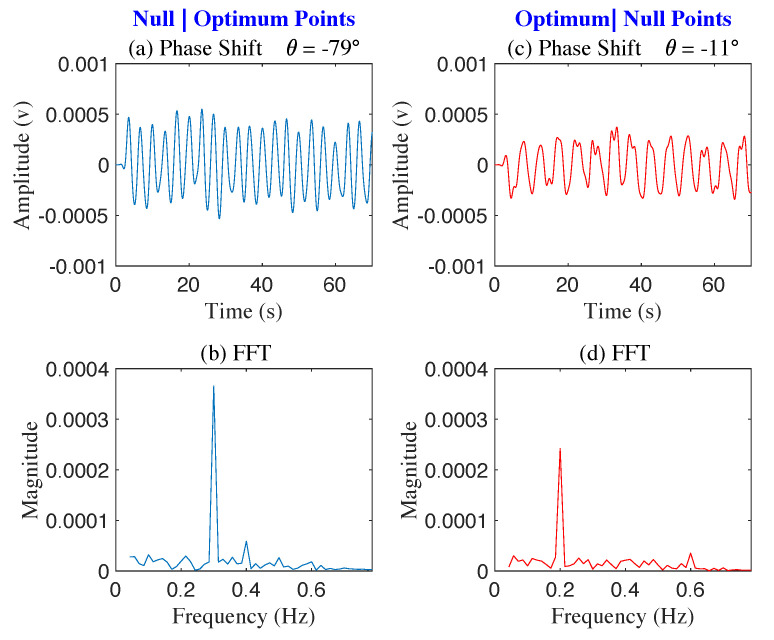
The figure shows two sets of demodulated signals, with $\theta_{ps}=-79^{\circ }$ (**a**,**b**) and $\theta_{ps}=-11^{\circ }$ (**c**,**d**). Robotic mover-1 0.3 Hz is at an optimum point, and mover-2 0.2 Hz is at a null point when $\theta_{ps}=-79^{\circ }$ (**b**). Mover-1 is at a null point, and mover-2 is at an optimum point when $\theta_{ps}=-11^{\circ }$ (**d**).

Figure 10 shows time and frequency domain signals for $\theta_{ps}=-79^{\circ }$ and $\theta_{ps}=-11^{\circ }$. At $\theta_{ps}=-79^{\circ }$, the frequency-domain plot indicates 0.2 Hz is in the null point position, and 0.3 Hz is in the optimum point position. At the phase shift value of $\theta_{ps}=-11^{\circ }$ frequency-domain plot indicates 0.2 Hz is in optimum point position and 0.3 Hz is in the null point position.

### 4.3. One Human Target and One Robotic Target Detection

**Figure 11:**
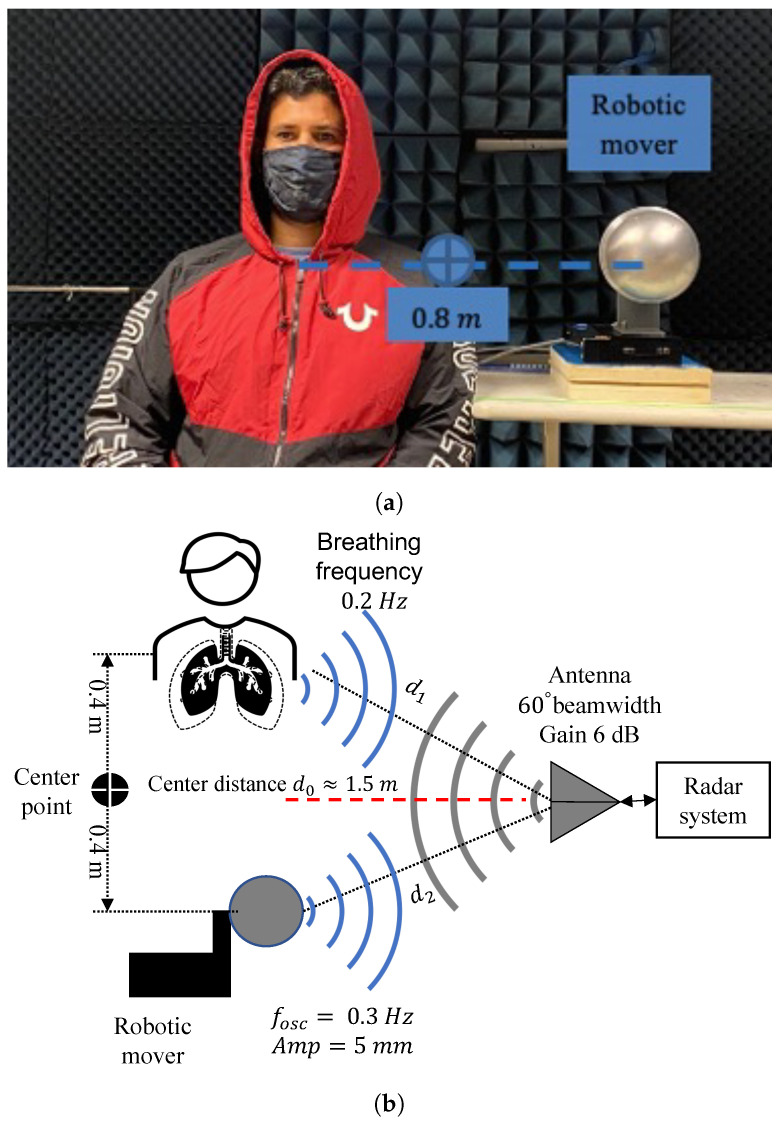
Photograph of the human subject and robotic movers placed facing radar antenna (**a**). The figure shows the experiment setup with the phase-sweeping radar, one robotic mover, and one human subject (**b**). The nominal distance between the radar and the targets is $d_{0}$ = 1.5 m. The distance between the targets is 0.8 m. The mover oscillates at 0.3 Hz frequency and 10 mm amplitude. The human subject breathes at 0.2 Hz.

The human subject and spherical shape robotic mover are placed facing the radar antenna at a distance of 1.5 m away from the radar antenna in Figure 11. The frequency oscillation of the robotic mover is 0.3 Hz, and the human subject breathing frequency is about 0.2 Hz using the metronome program as a breathing pacer. The center-to-center distance 0.8 m between the subject and robotic mover was chosen to represent the minimum distance between two humans shoulder-to-shoulder.

**Figure 12:**
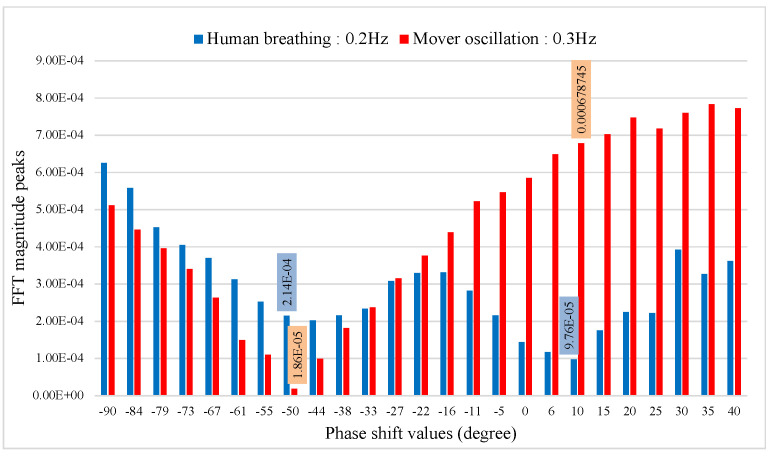
The figure shows the demodulated signals reflected from the two targets, with phase sweeping between $-90^{\circ }$ to $40^{\circ }$. The mover 0.3 Hz is at a null point, and the human target 0.2 Hz is near an optimum point when $\theta_{ps}=-50^{\circ }$. The mover is near an optimum point, and the human target 0.2 Hz is at a null point when $\theta_{ps}=10^{\circ }$.

**Figure 13:**
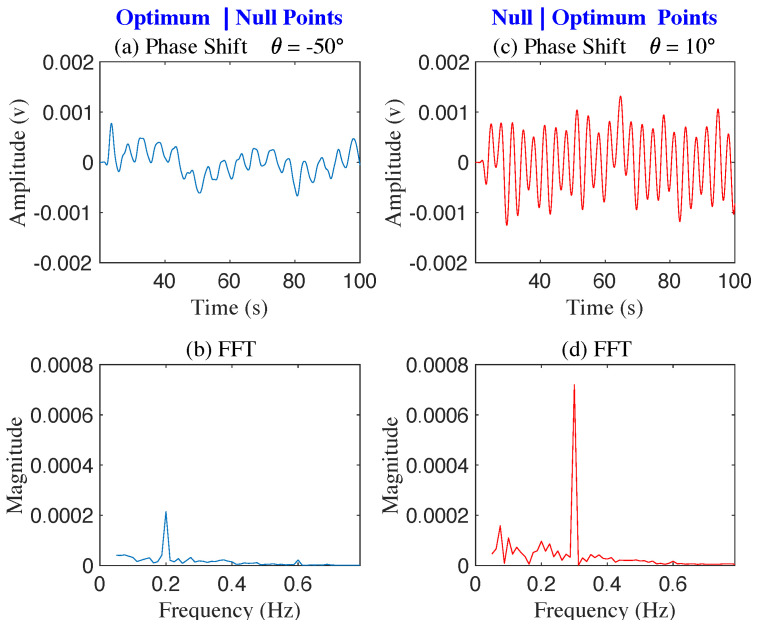
The figure shows two sets of demodulated signals, with $\theta_{ps}=-50^{\circ }$ (**a**,**b**) and $\theta_{ps}=-10^{\circ }$ (**c**,**d**). The mover 0.3 Hz is at at a null point, and the human subject 0.2 Hz is near an optimum point, when $\theta_{ps}=-50^{\circ }$ (**b**). The mover is near an optimal point, and the human subject is at a null point, when $\theta_{ps}=10^{\circ }$ (**d**).

Figure 12 shows the FFT of the baseband signal output for phase shift values from $-90^{\circ }$ to $40^{\circ }$. At phase shifter angle $-50^{\circ }$, the baseband signal of the mover at 0.3 Hz is suppressed to the null point and the optimum point for the mover appears at around $40^{\circ }$ as expected. While null/optimum positioning is more complex for a human subject, due to breath-to-breath amplitude variations and human subject likely shifting position slightly during measurements, it is still possible to identify minimum amplitude at $10^{\circ }$. Time and frequency domain plots for the phase shift of $-50^{\circ }$ to $10^{\circ }$ confirm that signal separation is indeed possible (see Figure 13).

The baseband signal can be recovered when the other is suppressed for the scenarios of multiple targets, regardless of their separation in the frequency domain. In case if there are more than two targets present, a single source signal can be recovered from multiple mix signals by initially suppressing it to identify the contribution from all other sources, which can then be adaptively filtered to recover the signal of interest. Number of sources that can be separated will be limited by the resolution of the phase shifter.

## 5. Conclusions

This paper presented a multi-target physiological detection and separation method suitable for single-antenna single-channel narrow band CWDRs. The technique employs the phase correlation detection principle and tunes the radar’s initial phase to align null and optimal points with the individual targets’ positions, thus achieving signal separation. Exploiting the wavelength-level spacing among null and optimal points, the demodulated signal can isolate nearly equidistant targets with diverse physiological spectra after primary signal processing steps such as FFT. Proposed phase tuning technique with FFT does not require significant hardware complexity not significant computational resources. However, this technique does rely on frequency separation of sources, thus for overlapping frequency spectra other signal processing technique such as MUSIC or empirical mode decomposition (EMD) may be explored in the future. We presented theoretical analysis, software simulations, full system implementation, and experiment evaluations with different mechanical and human target combinations. All show the system’s effectiveness in separating two closely spaced targets within a single antenna beam. Since human targets are likely to present different surface configurations and breathing dynamics, and thus different initial phase even at the same radar range, this method is promising for separation of physiological signals from multiple individuals. In the future, we plan to assess the performance of the phase correlation approach with higher number of targets, thus expanding its application into domains such as occupancy counting.

## Data Availability

The data that support the findings of this study are available from the corresponding author upon reasonable request.
